# Using the Assembly Time as a Tool to Control the Surface Morphology and Separation Performance of Membranes with a Tannic Acid–Fe^3+^ Selective Layer

**DOI:** 10.3390/membranes14060133

**Published:** 2024-06-06

**Authors:** Hluf Hailu Kinfu, Md. Mushfequr Rahman, Erik S. Schneider, Nicolás Cevallos-Cueva, Volker Abetz

**Affiliations:** 1 Helmholtz-Zentrum Hereon, Institute of Membrane Research, Max-Planck-Straße 1, 21502 Geesthacht, Germany; hluf.kinfu@hereon.de (H.H.K.); erik.schneider@hereon.de (E.S.S.); nicolas.cevallos-cueva@hereon.de (N.C.-C.); volker.abetz@hereon.de (V.A.); 2 Institute of Physical Chemistry, University of Hamburg, Martin-Luther-King-Platz 6, 20146 Hamburg, Germany

**Keywords:** metal–polyphenol network membranes, polyphenol, tannic acid–metal ion coordination, TFC membranes, assembly time

## Abstract

Thin-film composite (TFC) membranes containing a metal–polyphenol network (MPN)-based selective layer were fabricated on a porous polyacrylonitrile support. The MPN layer was formed through coordination-based self-assembly between plant-based tannic acid (TA) and an Fe^3+^ ion. For the first time, we demonstrate that TFC membranes containing TA-Fe^3+^ selective layers can separate small organic solutes in aqueous media from equimolar mixtures of solutes. The effect of the assembly time on the characteristics and performance of the fabricated selective layer was investigated. An increase in the assembly time led to the formation of selective layers with smaller effective pore sizes. The tannic acid–Fe^3+^ selective layer exhibited a low rejection towards neutral solutes riboflavin and poly(ethylene glycol) while high rejections were observed for anionic dyes of orange II and naphthol green B. Permeation selectivities in the range of 2–27 were achieved between neutral and charged dyes in both single- and mixed-solute experiments, indicating the significant role of Donnan exclusion and the charge-selective nature of the membranes. The rejection efficiency improved with an increasing assembly time. Overall, this study demonstrates that the assembly time is a vital casting parameter for controlling the permeance, rejection and selectivity of thin-film composite membranes with a tannic acid–Fe^3+^ selective layer.

## 1. Introduction

Selectivity severely restricts the scope of separations that can be achieved through membrane-based processes [1]. Engineering membranes for molecular separation remains a challenge. An increase in the selectivity tends to significantly decrease the permeability while increasing the cost and energy consumption. Available commercial membranes remove all solutes above a given size [2]. “Chemoselective” membranes that combine size-based selectivity with a solute–functional group differentiation capability can enable researchers to design membranes for customer applications with huge potential in the separation and purification process [3]. Although there are some attempts at separating similar-sized components through electrostatic contribution, successful separation of the desired components from one another still requires further work on the manipulation of the membrane chemistry [2]. In recent years, efforts have been devoted to developing membranes capable of regulating the permeation and rejection of small molecules of similar sizes through solute–membrane interaction in addition to size-based fractionation [3,4,5,6,7,8].

Thin-film composite (TFC) membranes are typically used for nanofiltration and reverse osmosis due to their high solute rejection properties [9]. TFC membranes are fabricated by constructing a dense selective layer over a porous polymer substrate, usually via interfacial polymerization (IP). However, often, such TFC membranes demonstrate rather poor selectivity between different solutes [9]. Therefore, the development of novel membranes that offer both high permeance and adequate selectivity is required to enhance the separation efficiency. Thin-film nanocomposite (TFN) membranes with incorporated nanomaterials have recently been developed [10,11,12,13] and shown to be promising materials in addressing these issues. TFN selective layers increase the surface hydrophilicity and membrane permeability by providing water pathways and enhance perm-selectivity [14,15]. However, the synthesis of TFN films is usually complicated, involving multi-step nanomaterial synthesis, separation and purification [15]. Scaling up the fabrication of TFN membranes also remains a concern [14]. More importantly, the fabrication of both TFC and TFN selective layers via interfacial polymerization involves the use of a significant amount of toxic and hazardous solvents, which makes the process less environmentally friendly [16,17,18]. Hence, there is a growing demand for the production of hydrophilic TFC membranes in an ecofriendly approach.

To date, there have been various approaches developed for the fabrication of sustainable TFC membranes. Yang et al. prepared biobased TFC membranes comprising a selective layer obtained from plant-based sources [19]. A green polyamide TFC membrane was fabricated via the interfacial polymerization of natural monomers, that is, genipin in the aqueous phase and priamine in a green organic solvent. Another TFC membrane was recently fabricated using building blocks of chitosan in the aqueous phase and 2,5-furandicarboxaldehyde (FDA) dissolved in the water-immiscible ecofriendly solvent eucalyptol [20]. Park et al. also reported a green way of fabricating a high-performance TFC membrane containing a crosslinked selective layer of priamine covalently bonded through Michael addition and Schiff base reactions to tannic acid [21]. These attempts demonstrate the prospects of breaking away from the toxic monomers besides exploring the use of green solvents. 

Tannic acid, a natural polyphenol, can be used for engineering new functional materials [22] such as TFC membranes [23]. Tannic acid’s phenolic groups carry negative charges, making them capable of easily chelating positively charged transition metal ions [24]. The formation of tannic acid–metal ion (TA-M^n+^) complexes arises from the strong coordination between phenolic hydroxyl groups and metal ions [25]. This coordination-driven self-assembly results in the formation of a metal–polyphenol nanofilm over a substrate surface [26], including the fabrication of thin films for membrane processes [25,27,28,29]. Compared with conventional water treatment membranes, metal–polyphenol network (MPN)-based membranes have several advantages: higher water flux with a comparative rejection of solutes, excellent resistance to fouling, high antimicrobial activity and efficient removal of organic contaminants [25]. More importantly, the metal–tannic acid membrane production is green and low-cost.

MPN has been used as a surface coating of ultrafiltration, nanofiltration and reverse osmosis membranes [15,27,30,31] and for the construction of the selective layer of a membrane [28,32,33]. Tannic-acid-containing membranes have shown excellent performance levels in several applications such as wastewater treatment [32], membrane fouling resistance enhancement [34,35], oil/water emulsion separation [29], desalination [36], separation of ions [33], trace organic contaminants’ removal [28] and organic solute separation [33]. Moreover, tannic-acid-based MPN layers can be coated using aqueous solutions, which is ecofriendly. In recent years, the optimization of the casting parameter of TFC membranes containing a tannic acid–metal ion layer has received a lot of attention. Co-deposition and layer-by-layer (LBL) self-assembly are the two widely used procedures for the fabrication of tannic acid–metal ion-containing membranes. Despite the fact that co-deposition enables the fast synthesis of TA–metal ion films [30], the rapid self-assembly makes the process uncontrollable [33]. The LBL technique allows a better control of the MPN layer coating synthesis and characteristics, which helps to significantly reduce the undesired defects of the selective layer of the membranes [37].

In a previous study [38], we investigated the influence of the TA-to-Fe^3+^ ratio on the surface porosity and filtration properties of the MPN selective layer containing TFC membranes. However, the impact of the assembly time on the structure and performance of TFC membranes containing TA-Fe^3+^ selective layers prepared by LBL remained to be explored. Accordingly, here, we performed a thorough study of the influence of the assembly time on the morphology and separation performance of TFC membranes containing TA-Fe^3+^ selective layers prepared by LBL. The development of membranes for effective solute–solute separation with excellent precision is still in its early phases [39]. In recent years, the development of membranes for the separation of organic solutes with dimensions of 0.5–5 nm has received a lot of attention as there is a lack of commercially available membranes to perform the task [6,7]. In this work, we demonstrate that controlling the assembly time of the formation of TA-Fe^3+^ coordination is an effective tool when performing such separation using TFC membranes containing TA-Fe^3+^ selective layers.

## 2. Experimental Section

### 2.1. Materials and Chemicals

The porous polyacrylonitrile (PAN) membrane support was prepared in-house at Helmholtz-Zentrum Hereon. Tannic acid (TA) (1701.2 g/mol), orange II (OR-) (350.32 g/mol), riboflavin (RB0) (376.36 g/mol) and naphthol green B (NGB3-) (878.46 g/mol) were commercially supplied by Sigma-Aldrich Chemie GmbH (Taufkirchen, Germany). FeCl_3_·6H_2_O was obtained from Alfa Aesar GmbH & Co. (Karlsruhe, Germany). Poly(ethylene glycol)s (PEGs) of average molecular weights (Mw) 200, 400, 600 and 1000 g/mol were purchased from VWR International GmbH (Darmstadt, Germany). Hydrochloric acid (HCl, 37%) and sodium hydroxide (NaOH) were supplied by Merck Biosciences GmbH (Darmstadt, Germany) and Sigma-Aldrich, respectively. All the chemicals were analytical-grade reagents and were used as received without further purification.

### 2.2. Membrane Selective Layer Synthesis

**Figure 1:**
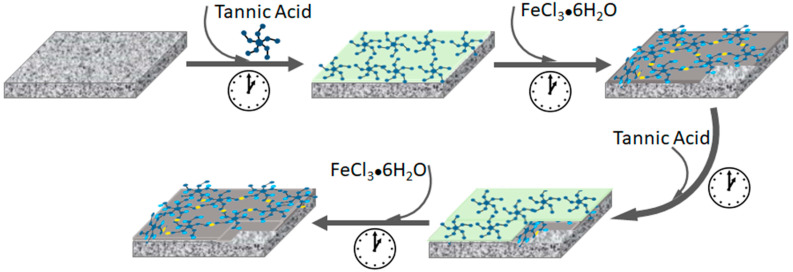
Schematic representation of the fabrication process of the membrane selective layer. TFC membrane containing TA-Fe^3+^ is fabricated by depositing tannic acid and iron aqueous solutions over a porous PAN support in a layer-by-layer technique.

The TA-Fe^3+^ TFC membrane synthesis procedure was reported in our previous work [38]. Briefly, the pristine PAN support was pre-soaked in milli-Q water before further use. Then, the PAN support was fixed to a custom-designed glass plate and clapped with a PTFE frame to ensure that the MPN layer was synthesized only on the top surface of the porous support. Next, the PAN support was subjected to 50 mL TA solution (0.02 w %) for a specified time. The TA-treated porous support was then rinsed with water. Afterwards, 50 mL metal ion solution (0.09 w % FeCl_3_·6H_2_O) was added for the same period to coordinate with the already adsorbed TA groups. The TA-Fe^3+^-coated PAN membrane was further rinsed with pure water and subjected to both TA and Fe^3+^ solutions in the same procedure in a layer-by-layer fashion to prepare a two-TA-Fe^3+^-layered thin film, as shown in Figure 1. The assembly time was varied to prepare a series of TFC membranes. The PAN support was exposed to both TA and Fe^3+^ solutions for the same duration, i.e., for 1 min, 2.5 min, 4 min or 6 min in each solution, and the corresponding fabricated TFC membranes are labeled MPN-1, MPN-2.5, MPN-4 and MPN-6, respectively.

### 2.3. Membrane Characterization

Fourier transform infrared spectroscopy (FTIR) using a Bruker Alpha (diamond-ATR unit) (Bruker, Karlsruhe, Germany) was used to analyze the functional groups and confirm the presence of TA in the TA-Fe^3+^ selective layer synthesized over the PAN support. The spectrum of each membrane was measured using 64 scans in the range of 400–4000 cm^−1^ with a resolution of 4 cm^−1^. Membrane morphological characteristics were characterized by scanning electron microscopy (SEM) (Merlin SEM, Zeiss, Oberkochen, Germany) using accelerating voltages of between 1.5 and 3 kV. Membrane samples were vacuum dried at 60 °C for 72 h before being sputter-coated with 1–1.5 nm platinum using a CCU-010 coating device (Safematic, Switzerland). For cross-sectional image analysis, the membrane samples were prepared by immersing and fracturing them in liquid nitrogen. SEM images based on material contrast using backscattered electrons were also obtained using an in-lens EsB detector. Samples of the membrane cross-section were prepared by argon ion milling at −120 °C and 4 kV for several hours. A precision argon ion beam milling etching and coating system PECS II (Gatan/AMETEK, Pleasanton, CA, USA) was used. The samples were coated with 4 nm carbon before measurement. The hydrophilic properties of the membranes were analyzed by measuring the water contact angle (WCA) using KRUESS Drop Shape Analysis System DSA 100 (FEI part of Thermo Fisher Scientific, Kawasaki, Japan). The investigations were performed in the sessile drop mode with 3 µL of water droplets at room temperature. The membrane surface zeta potential was measured by a SurPASS 3 electrokinetic analyzer (Anton Paar, Graz, Austria) using a background electrolyte solution containing 1 mmol NaCl. The zeta potential measurements were taken in the pH range of 5–8 at room temperature. Energy-dispersive X-ray spectroscopy (EDX) was performed to determine and compare the elemental compositions of the membrane top layers using Extreme as the primary and X-max 150 as the secondary EDX detector (Oxford Instruments, Abingdon, UK). The EDX analysis was performed at a working distance of 5.6 mm, a constant magnification of 5 kx and an acceleration voltage of 1.5 kV.

### 2.4. Evaluation of Membrane Performance

The membrane filtration performance was evaluated with pure water permeance and solute rejection experiments. All experiments were performed in the dead-end filtration mode using a 2.13 cm^2^ membrane active area. The membranes were compacted for at least 3 h at 4 bar transmembrane pressure. Then, the pure water permeance, $P_{w}$ (L·m^−2^·h^−1^·bar^−1^), was measured and computed using the following equation:

(1) $$P_{w}=\frac{V}{A*∆t*∆P}$$

where V (L) is the volume of permeate collected, A (m^2^) is the effective area of the membrane, ∆t (h) is the filtration time and ∆P (bar) is the applied transmembrane pressure.

Characterization of the membrane rejection performance was carried out using a stirred test cell from Millipore (EMD Millipore XFUF07601) (Merck Millipore, Darmstadt, Germany) at a stirring speed of 350 rpm in the dead-end filtration mode. The effective membrane area of the test cells was reduced to the desired size with an in-house-made reduction ring. PEG, dyes and Na_2_SO_4_ were used as solutes for the investigation. All rejection tests were performed at 3 bar transmembrane pressure. The concentrations in the feed, permeate and retentate from the filtration test using PEG (1 g·L^−1^) were analyzed using gel permeation chromatography (GPC) (VWR-Hitachi 2130 pump, Hitachi, Darmstadt, Germany). The samples from the rejection test of dyes (feed concentration of 0.1 mM) were analyzed by a UV-Vis spectrophotometer (GENESYS 10S, Thermo Scientific). The salt rejection performance of the membranes was measured with a 1 g·L^−1^ aqueous solution of Na_2_SO_4_, and samples were analyzed using ion chromatography (Dionex ICS600, Thermofischer Scintific Inc., Waltham, MA, USA).

To investigate the separation behavior of the synthesized membranes towards mixed solutes, 1:1 molar mixtures of the dye solutions of 0.1 mM total feed concentration were filtered with the aforementioned filtration setup. The recorded feed, permeate and retentate samples were analyzed using UV-Vis spectrophotometry.

The observed rejection was then evaluated as follows:

(2) $$\;R\;\left(\%\right)=\left(1-\frac{C_{p}}{(C_{f}+C_{r})/2}\right)*100$$

where R is solute rejection, and $C_{p}$, $C_{f}$ and $C_{r}$ are the concentrations of the permeate, feed and retentate solutions in mg·L^−1^, respectively. The concentrations before and after permeate collection were measured. During the test, the feed-side concentration gradually changes due to the passage of permeate solution across the membrane active layer. However, the change in the concentration is minor as the feed volume is huge compared to the permeated volume of the test solution. The average of the feed and retentate concentrations is taken into account for consideration of the small change in feed-side concentration in the dead-end filtration mode. The arithmetic mean of $C_{f}$ and $C_{r}$ is an approximation usually performed to measure solute retention [40]. The membrane selectivities during the rejection tests of both single solutes and mixed solutes are calculated using

(3) $$Selectivity=\frac{100-R_{1}(\%)}{100-R_{2}(\%)}$$

where subscripts 1 and 2 represent solute 1 and solute 2, respectively. 

In order to evaluate the fouling resistance potential of the TA-Fe^3+^ TFC membranes, the antifouling properties of the fabricated membranes were further assessed with the filtration of humic acid (HA) solution. A humic acid feed solution of 100 ppm at a pH of ~7, adjusted with HCl and NaOH, was used for these experiments. The experiments were conducted according to the following procedure. First, pure water flux tests were performed for 1 h to determine the initial water flux ($W_{i}$). Then, the feed solution was replaced with HA solution for the fouling studies for 1.5 h. After HA solution filtration, the fouled membrane samples were taken out and rinsed with pure water. Finally, the pure water flux of the fouled membranes ($W_{o}$) was measured again for 1 h. The procedure was repeated again for a second cycle test. All experiments were performed at 3 bar transmembrane pressure. Equations (4) and (5) were used to compute the water flux (W) and the flux recovery ratio (*FRR*) of the analyzed samples, respectively.

(4) $$W=\left(\frac{V}{A*∆t}\right)$$

(5) $$FRR=\left(\frac{W_{i}}{W_{o}}\right)*100$$

## 3. Results and Discussion

### 3.1. Influence of Assembly Time on the Membrane’s Physicochemical and Morphological Properties

**Figure 2:**
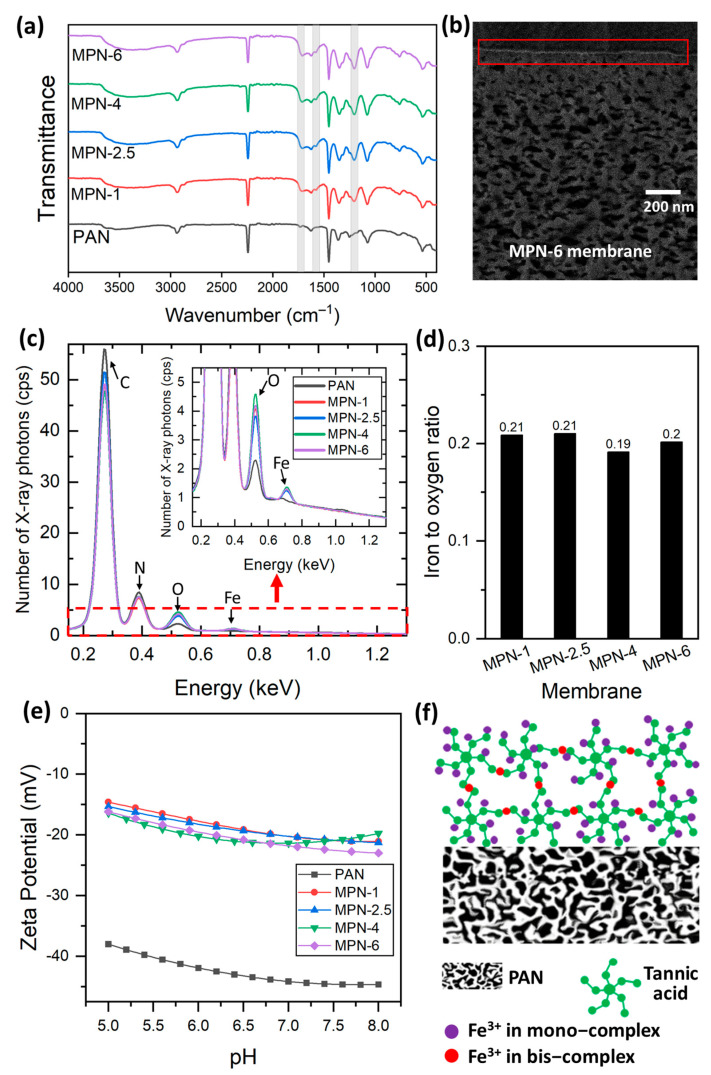
(**a**) FTIR spectra of the PAN and TA-Fe^3+^ membranes fabricated at different assembly times. (**b**) Backscattered electron SEM image of a TA-Fe^3+^ TFC membrane sample showing a continuous, thin, Fe-rich selective layer deposited over the porous substrate. Elemental analysis of the PAN support and the TFC membranes: (**c**) EDX spectra and (**d**) Fe/O ratio of the TFC membranes. Inset in (**c**) displays a zoomed−in figure showing the peaks for oxygen and iron. (**e**) Surface zeta potential as a function of pH for the PAN support and TA-Fe^3+^ membranes fabricated at different assembly times. (**f**) A schematic illustration of the TA–metal ion coordination for thin-film formation by the LBL technique. MPN-1, MPN-2.5, MPN-4 and MPN-6 represent TA-Fe^3+^ TFC membranes fabricated at 1 min, 2.5 min, 4 min and 6 min of assembly time, respectively.

The general procedure of the TFC membrane fabrication followed in this work is shown in Figure 1 while the parameters used for casting the TA-Fe^3+^ selective layers on top of a PAN porous support are listed in Table 1. Apart from the assembly time, all the other parameters were kept unchanged while coating the TA-Fe^3+^ selective layers of the TFC membranes. Table 1 shows the four TFC membranes prepared in this work, which all have double TA-Fe^3+^ layers. It should be mentioned here that the permeance through the membranes decreases with the increase in the number of deposited TA-Fe^3+^ layers [38]. The goal of this work was to achieve homogeneous TA-Fe^3+^ layers with the minimum possible number of coating cycles and to control the permeance by varying the assembly time. At least two coating cycles were required to achieve a homogeneous TA-Fe^3+^ layer using 0.1176 mM TA and 3.33 mM FeCl_3_.6H_2_O. In what follows, the TFC membranes are addressed by the assigned membrane name in Table 1, each corresponding to the assembly time used for the coating of the TA-Fe^3+^ selective layers. FTIR spectroscopy was used to investigate the deposition of TA-Fe^3+^ selective layers on the microporous PAN support. As illustrated in Figure 2a, the PAN substrate shows its absorption band for the nitrile group (C≡N stretching vibration) at 2242 cm^−1^ [41]. The bands at 2937 and 1453 cm^−1^ correspond to the CH_2_ vibration peak and the bending of the C-H bond in CH_2_, respectively. Conversely, the membranes containing a TA−Fe^3+^ selective layer show characteristic peaks different to those observed in the pristine PAN support. A new peak at 1202 cm^−1^ is ascribed to the C-O stretching vibration while the absorption peak at 1575 cm^−1^ is attributed to the C=C stretching vibration bond from the aromatic groups of TA. Representative peaks for the C=O stretching vibration from the ester groups of tannic acid are also shown at 1713 cm^−1^ [27]. The broad FTIR spectrum between 3100 and 3700 cm^−1^ is attributed to the phenolic -OH groups of TA. Generally, these results confirm the successful coating of the TA-Fe^3+^ layer on top of the porous PAN support. The TA-Fe^3+^ layer at the top of the microporous PAN support was also visible in a backscattered electron SEM image as a thin bright line owing to presence of electron-rich Fe in the layer. As an example, the backscattered electron SEM image of the TFC membrane sample synthesized at 6 min of assembly time (MPN-6) is provided in Figure 2b.

Figure 2c shows the EDX spectra obtained at the surfaces of the PAN support and the TFC membranes. As expected, the PAN support was composed of a significant amount of carbon and nitrogen. A small amount of oxygen is also visible in the EDX spectrum of PAN, which is in accordance with the –OH peak observed in the FTIR spectrum of PAN (Figure 2a). The EDX spectrum of the TFC membranes shows the elements that are present in the thin TA-Fe^3+^ layer as well as the PAN support owing to the penetration depth of the high-energy electron beam used to excite the samples. The EDX analysis confirmed the presence of Fe in the TFC membranes. The content of oxygen in the TFC membranes was significantly higher compared to the PAN support. The iron-to-oxygen ratio in the TA-Fe^3+^ TFC membranes was computed from the integrated area of the peaks (after correcting the peak area by subtracting that of PAN). All membranes presented ~0.2 for the Fe-to-O ratio, as shown in Figure 2d. The –OH containing pyrogallol and catechol moieties in TA acts as a bidentate ligand for the metal ion center during metal–polyphenol self-assembly to form mono, bis and/or tris complexes [26,42]. The formation of the complexation state is dictated by several factors including the pH, metal-to-ligand ratio and concentration of casting solutions [26,43,44]. Previously, we provided the possible formation mechanism of the TA-Fe^3+^ selective layer through the LBL technique [38]. The protocol involves a four-step procedure of TA adsorption and TA–metal ion coordination, which leads to the formation of both mono- and bis-complex states. The theoretical Fe/O ratios of the three different complexation states (calculated from the chemical structure considering that a TA molecule contains 46 oxygen, out of which 25 are from –OH groups) are listed in Table S1 of the Supporting Information. The experimentally observed iron-to-oxygen ratios of the TFC membranes lie between the theoretical Fe/O ratios of the mono complex (0.27) and bis complex (0.14) for all TA-Fe^3+^ membranes. This proves that the TFC membranes contain a mixed mono-and-bis-coordinated complexed TA-Fe^3+^ layer, which is consistent with the findings of our previous study [38]. Figure 2d demonstrates that the assembly time has no significant influence on the coordination state.

**Figure 3:**
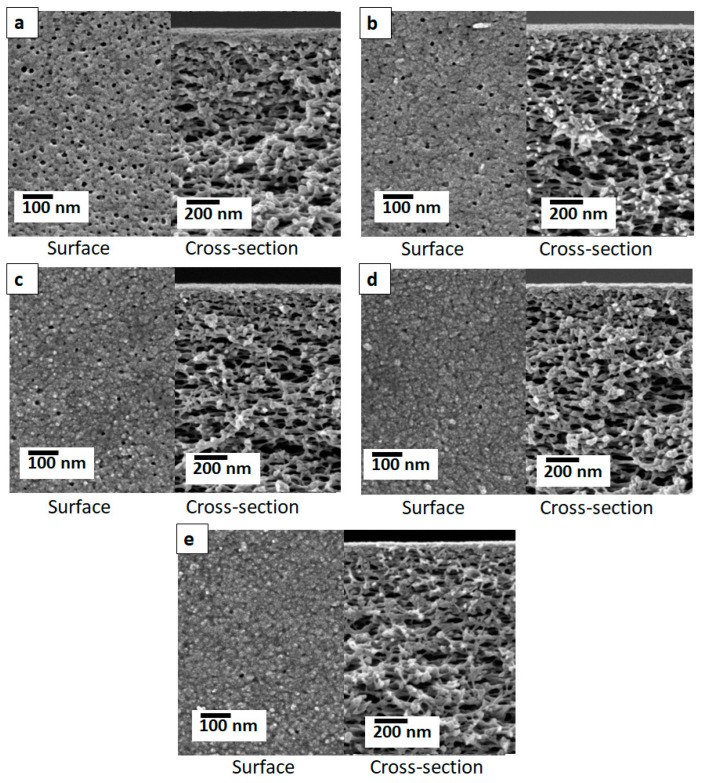
Top surfaces and corresponding cross-sectional SEM images of (**a**) the PAN support, and TA-Fe^3+^ TFC membranes synthesized at (**b**) 1 min (MPN-1), (**c**) 2.5 min (MPN-2.5), (**d**) 4 min (MPN-4) and (**e**) 6 min (MPN-6) of assembly time.

The streaming zeta potentials at the surfaces of the membranes were measured to evaluate the surface charge property. Figure 2e demonstrates that the TFC membranes had negative zeta potentials in the investigated pH range, owing to the acidic nature of the galloyl groups in tannic acid [26]. Polyphenols possess numerous dissociable hydroxyl groups in their structure. The zeta potential profiles of the fabricated membranes showed a gradual increase with the decrease in pH. The PAN support exhibited a negative surface charge. However, the negative surface charge of the bare PAN membrane was weakened after the TA-Fe^3+^ coating. Variation in self-assembly time has no significant influence on the surface zeta potential of the membranes. It is well-established that PAN and other polymeric surfaces with no dissociable groups demonstrate a negative zeta potential owing to the preferential adsorption of –OH groups of water compared to hydronium ions [45]. Figure 2e implies the TA-Fe^3+^ coating hinders the preferential adsorption of –OH groups of water at the surface to some extent. The lower negative surface charge of the TFC membranes containing a TA-Fe^3+^ selective layer compared to that of the PAN support is consistent with the findings of our previous study [38]. While there was no significant influence on the TA-Fe^3+^ coordination state and surface charge (Figure 2), a critical influence of the assembly time on the surface morphology of the TFC membranes was observed (Figure 3). A SEM image of the PAN support is shown in Figure 3a. The pristine PAN contains a smooth and porous top surface. Figure 3b–e present the top surface and cross-sectional SEM images of the TFC membranes synthesized at various assembly times. In comparison to the PAN support, the top surface of the fabricated TFC membranes demonstrates a significant decrease in porosity due to the MPN-layer coating. From Figure 3, it is also evident that the MPN-layer coating does not penetrate the porous support, and the porosity at the spongy support layer of the membranes remains unchanged. A careful comparison of Figure 3b–e shows that the surface porosity of the TFC membranes gradually decreases with the increase in assembly time. In other words, the top layer becomes denser with the increase in the assembly time. The EDX analysis demonstrated that there is no significant influence of the assembly time on the coordination state. For the studied assembly times (i.e., 1–6 min), it was not possible to decipher any significant increase in the thickness of the TA-Fe^3+^ selective layers of the TFC membranes from the cross-sectional SEM images (Figure 3b–e). The LBL coating protocol allowed for controlled deposition of TA-Fe^3+^ on the surface of the PAN layer. TFC membranes with similar surface charges but different surface porosities were obtained by varying the assembly time.

### 3.2. Filtration Performance of the Membranes

#### 3.2.1. Water Contact Angle and Pure Water Permeance of the Fabricated Membranes

**Figure 4:**
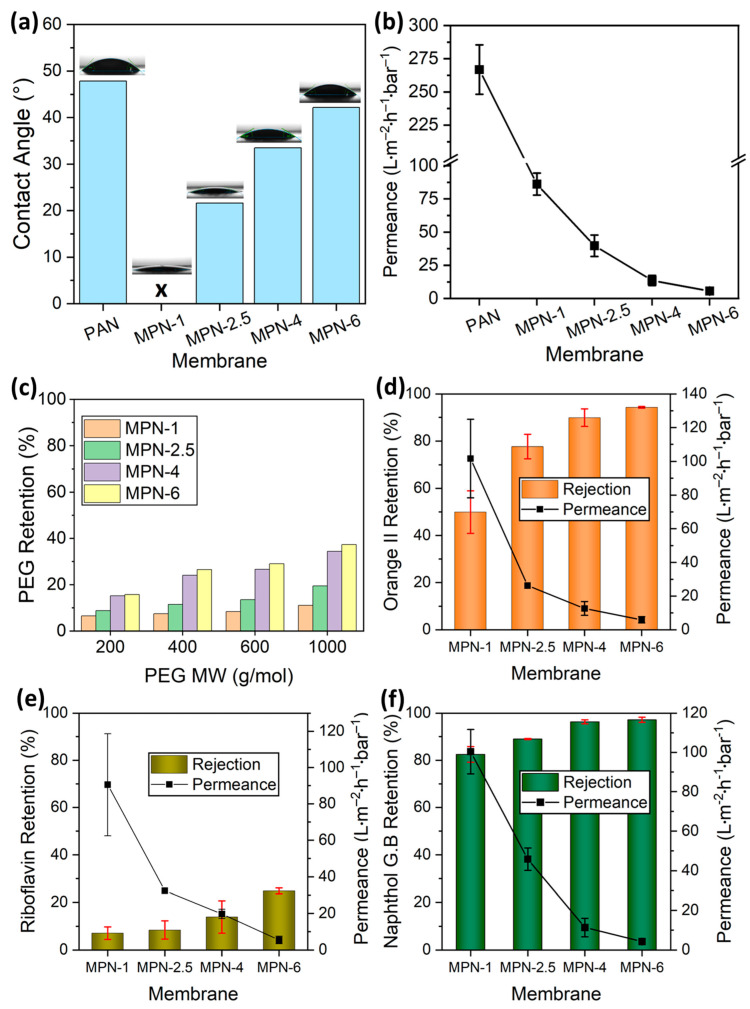
(**a**) Water contact angle and (**b**) pure water permeance of the PAN support and TA-Fe^3+^ membranes fabricated at different assembly times. Retention of a single solute—(**c**) poly(ethylene glycol) (neutral), (**d**) orange II (–1 charge), (**e**) riboflavin (neutral) and (**f**) naphthol green B (–3 charge)—by the TA-Fe^3+^ membranes as a function of the assembly time of the membranes. The solid line is added to guide the eye.

The water contact angle (WCA) analyses of the polyacrylonitrile support and the TFC membranes are shown in Figure 4a. The pristine support exhibited a WCA of around 48°. The WCA of the PAN membrane declined after the TA-Fe^3+^ coating. It is important to mention here that the water contact angles are influenced by both the chemistry and porosity of the membranes. The TA-Fe^3+^ layers, owing to the presence of hydroxyl groups, are substantially more hydrophilic than the PAN. The hydrophilic nature of tannic acid [46] tends to decrease the WCA of the TFC membranes compared to that of the PAN membrane. On the other hand, for TA-Fe^3+^-containing membranes, the loss of porosity tends to increase the WCA of the TFC membranes. Among the TFC membranes, the one fabricated using a one-minute assembly time (MPN-1) had the highest surface porosity. Therefore, it had the lowest WCA. In fact, the WCA was too low to measure. The contact angle increased from 22° to 42° when the assembly time was lengthened from 2.5 min to 6 min. The dynamic water contact angles of the membranes are provided in Figure S2 of the Supporting Information.

The pure water permeance (PWP) of the support was around 266 ± 17 L·m^−2^·h^−1^·bar^−1^. Figure 4b presents the PWPs of the synthesized TFC membranes. As expected, the permeance dropped significantly after the deposition of the TA-Fe^3+^ selective layer. This indicates a decrease in pore size and porosity compared to the porous pristine PAN surface. Furthermore, the membrane surface chemistry was altered after the metal–polyphenol network coating. A sharp decrease in water permeance through the TFC membranes can be observed as the assembly time is increased from 1 to 6 min (Figure 4b). The water permeance decreased owing to the lower porosity of the resulting membranes, as depicted in the SEM images presented in Figure 3. The pure water flux of the assembled membranes decreased by more than 6-fold when the assembly time was increased from 1 min to 4 min. The membrane synthesized at 6 min (MPN-6) showed a PWP of 5.6 L·m^−2^·h^−1^·bar^−1^. The salt rejection performance of the membranes was also investigated. The retention of Na_2_SO_4_ increased from 32% to 97% with the increase in assembly time from 1 to 6 min (Figure S1, Supporting Information). Overall, the fabricated membranes displayed a high Na_2_SO_4_ retention performance in comparison to other tannic acid–metal ion membranes published in the literature, as shown in Table S2 of the Supporting Information.

#### 3.2.2. Organic Solute Retention Performance

Figure 4c–f show the observed organic solute rejection performance of the prepared TFC membranes. The neutral PEG and riboflavin had rather high permeances through the TFC membranes, while the anionic dyes orange II and naphthol green B were highly retained. PEG retention tests are the most commonly employed experiment to investigate the pore sizes of such membranes. We investigated the retention of PEG with molecular weights of 200, 400, 600 and 1000 g/mol by the TFC membranes. In every case, the retention of PEG of a specific molecular weight increased with the assembly time (Figure 4c), indicating the decline in the pore size of the selective layer. Hence, the findings of our morphological investigations based on SEM (Figure 3) and PEG retention were in agreement with each other. The retention rates of PEG of 1000 g/mol molecular weight by the TFC membranes prepared by using 1, 2.5, 4 and 6 min assembly times were 11%, 20%, 34% and 37%, respectively. These results confirm that although the pore size and porosity of the selective layers of the TFC membranes gradually decrease with the assembly time, each of the membranes has sufficiently large pores to allow the permeation of PEG with a molecular weight of 1000 g/mol. Hence, as expected, the retention of the neutral molecule riboflavin (molecular weight 376.76) is rather low, in the range of 7 to 25% (Figure 4e). It is worth mentioning here that for the TFC membranes prepared using 1, 2.5, 4 and 6 min assembly times, the retention rates of riboflavin were 7, 8, 14 and 25%, while the retention rates of PEG with a molecular weight of 400 g/mol were 7, 12, 24 and 27%, respectively. Hence, the retention rates of neutral molecules are in good agreement with one another. Figure 4c,e show a similar trend of rejection of neutral molecules (i.e., PEG and riboflavin). With the increase in assembly time, the rejection gradually increases due to a loss of surface porosity of the membranes. Figure 4d,f also show an increase in rejection of the anionic molecules (i.e., orange II and naphthol green B) with an increase in the assembly time. Unlike the neutral molecules, the anionic dyes orange II and naphthol green B were highly retained by the TFC membranes. For example, the retention rates of orange II and naphthol green B were 50% and 83%, respectively, for the TFC membrane prepared at 1 min of assembly time (Figure 4d,f). The naphthol green B retention rate was 97% while that of orange II was 94% when the assembly time was increased. The rejection of orange II was significantly improved when the coordination proceeded to four minutes. Surface porosity decreased only up to an assembly time of four minutes and led to an improved solute removal performance. An assembly time beyond four minutes resulted in a decline in water permeance by more than two-fold whilst no substantial variation in the anionic dyes’ rejection was exhibited. Therefore, a compact selective layer can be formed within just a few minutes. Essentially, the self-assembly of polyphenols and metal ions occurs instantly [27,47]. However, as TA contains numerous –OH groups, complete coordination between the two components is unlikely to occur. Hence, a loose network with elevated flux and relatively lower solute rejection is obtained at a short assembly time. Coordination proceeds through the involvement of more functional groups with the prolongation of the assembly time. However, the ideal selectivity of the orange II/naphthol green B pair does not change significantly with the assembly time of the studied membranes. For these anionic dyes, the ideal selectivities ranged between 2 and 3 (Table 2). On the other hand, the ideal selectivity of riboflavin/naphthol green B during a single-solute retention test increased from 5 to 27 when the assembly time increased from 1 min to 6 min (see Table 2), indicating a high permeation of neutral molecules while retaining charged groups. Similarly, the riboflavin/orange II selectivity increased from 2 to 13 with the increase in the assembly time. These results indicate that the fabricated membranes are suitable for charge-based organic/organic separation. The retention rates of the dye molecules are ordered riboflavin < orange II < naphthol green B for all membranes. Both size sieving and electrostatic exclusion play an important role in the selectivity and retention of organic solutes [48,49]. Therefore, it seems that the high retention of orange II as well as naphthol green B constitutes a combination of steric and Donnan effects. Although riboflavin and orange II have similar molecular sizes and molecular weights, the rather high retention rate of orange II suggests that Donnan exclusion predominantly affects the separation mechanism for the partitioning of solutes at the membrane–solution interface. The negatively charged TA-Fe^3+^ membrane surface (Figure 2e) rejects solutes that are smaller than the membranes’ molecular weight cut-off for neutral molecules, which is due to electrostatic repulsion. This demonstrates the high uncharged dye/charged dye selectivities of the membranes. The discrepancy in retention rates between the anionic dye molecules originates from their molecular sizes and charges. Orange II (350.32 g/mol) is a smaller monovalent dye (with –1 charge) compared to the bigger naphthol green B (878.46 g/mol) (with –3 charge) in aqueous solution. Therefore, the membrane–solute electrostatic repulsive interaction is significant for naphthol green B. Moreover, for the relatively porous membranes fabricated at a short assembly time, the small molecules of orange II can permeate through the TA-Fe^3+^ active layer more easily compared to naphthol green B. This aligns with the observed high selectivity for the MPN-1 membrane obtained with a 1 min assembly time. However, as the assembly time is increased, dense membranes are fabricated, restricting the permeation of orange II through the TFC membranes, which leads to a comparable retention of the two anionic dyes at a long assembly time. 

#### 3.2.3. Mixed-Solute Retention

**Figure 5:**
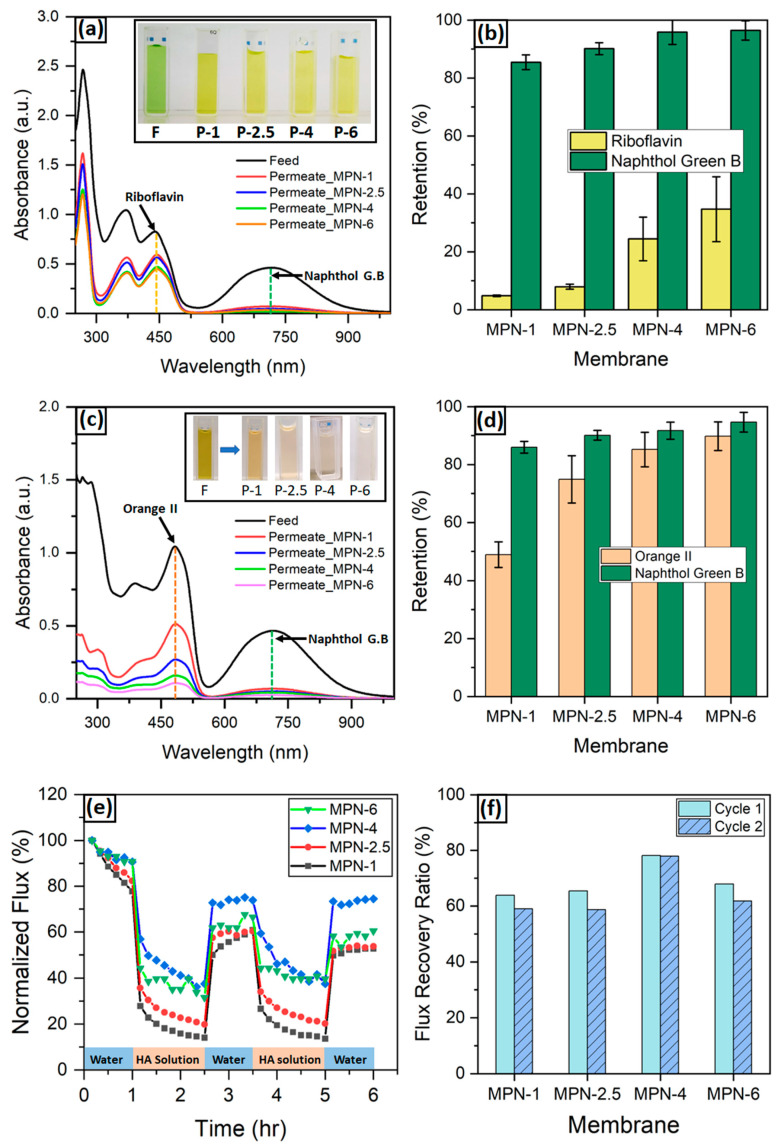
(**a**) UV-vis absorption spectra of the feed and permeate solutions and (**b**) retention of the 1:1 molar mixture of riboflavin and naphthol green B dyes, with peaks at 444 nm and 715 nm, respectively, through the TA-Fe^3+^ membranes synthesized at different assembly times. Inset: photographic images of the mixed dye solutions before (feed, F) and after (permeate, P) filtration experiments. Numerical values of 1, 2.5, 4 and 6 represent the assembly time (min) durations at which the membranes were fabricated by exposing the PAN surface to the TA and FeCl_3_·6H_2_O solutions. (**c**) UV-vis absorption spectra of the feed and permeate solutions and (**d**) retention of the dyes in the OR-/NGB3- system of the 1:1 molar mixture of orange II and naphthol green B dyes, with UV-vis absorption peaks at 482 nm and 715 nm, respectively, through the TA-Fe^3+^ membranes synthesized at different assembly times. (**e**,**f**) Evaluation of the antifouling properties of the metal–phenolic selective layers; (**e**) normalized water and HA solution flux of the TA-Fe^3+^ membranes and (**f**) flux recovery ratios of the synthesized membranes. The investigation was performed by first recording the PWP of a membrane sample for 1 h followed by HA solution filtration for 1.5 h. Then, the membrane was rinsed with pure water to remove fouled HA molecules, after which the PWP of the fouled membrane was measured for one hour again to determine the FRR. This process was repeated for two cycles.

The ideal selectivity calculated from the study of the permeation behavior when using feed solutions containing only single dyes demonstrated the potential of using the prepared TFC membranes to separate one dye from another. To check the validity of these data in order to determine the separation performance, it is important to study the permeation behavior using feed solutions containing mixtures of dyes. Coexistent substances in a feed solution can influence the retention and selectivity of solutes by membranes. For instance, plasticization of the membrane active layer by one solute may lead to a decrease in the rejection of all solutes, while adsorption of solutes into the pores may lead to constant variation in the rejection of all components [3]. Equimolar mixtures of dye solutions of riboflavin and naphthol green B (RB0/NGB3-) as well as orange II and naphthol green B (OR-/NGB3-) were filtered through the fabricated TFC membranes. It is worth mentioning here that in our previous work [38], we reported that the anionic OR- and NGB3- did not absorb on TFC membranes containing TA-Fe^3+^ selective layers and a PAN support layer even after immersing the membranes in the dye solutions for 24 h. Figure 5a,b display the effect of the assembly time on the retention and separation of an RB0/NGB3- mixture. The UV-vis spectra of the feed and permeate solutions are plotted in Figure 5a. While the peaks for RB0 strongly appear in the UV-vis spectra of the permeate solutions, the NGB3- peak is hardly visible. Hence, as expected from the study of feed solutions containing a single dye, from the equimolar RB0/NGB3- mixture, the TFC membranes allowed RB0 to permeate rather easily and highly retained NGB3-. That is visible even in the photographs of the feed and permeate solutions (Figure 5a), which show the colors of the solutions turning from green to yellow after permeating through the TFC membranes. The intensities of the UV-vis spectra at 444 nm and 715 nm were used to determine the retention of RB0 and NGB3- from the mixture, respectively (Figure 5b). Figure 5c,d show that the TFC membranes can also separate OR- and NGB3- from a mixed solution, as anticipated from the study of the solutions containing single dyes. A color change is visible for the solution that permeated through the TFC membranes prepared using a 1 min assembly time (Figure 5c). As evident from the corresponding UV-vis spectrum (Figure 5c), this TFC membrane retains NGB3- while it allows a large portion of OR- from the mixture to permeate through. As the pore size decreases (with an increasing assembly time), the TFC membranes not only highly retain NGB3- from the mixture but also OR-. Figure 5c shows the color of the permeate solutions gradually fade away with an increasing assembly time of the TFC membranes, while the intensity of OR- in the UV-vis spectra of the corresponding permeate solutions also decreases gradually. From the retention plot in Figure 5d, it is clear that the difference in retention rates between OR- and NGB3- by the TFC membranes gradually narrows with an increasing assembly time. This is reflected in the real selectivity values (mixed solutes) of the OR-/NGB3- pair (Table 2). In general, there is a trade-off between the permeance and selectivity, i.e., the membranes with high permeances suffer from low selectivities and vice versa. However, the separation of OR- and NGB3- from their equimolar mixtures by the studied TFC membranes does not follow this trade-off. The MPN-1 TFC membrane prepared at a 1 min self-assembly time has the highest OR-/NGB3- selectivity (3.6). Owing to its large porosity, it also has the highest permeance among the four studied TF membranes. However, the TFC membranes follow the trade-off between permeance and selectivity for the RB0/NGB3- pair. Table 2 shows that, in general, there are some deviations of the real selectivity (mixed solutes) from the ideal selectivity (single solutes). For example, the studies of single-dye retention led us to a significant overestimation of the RB0/NGB3- selectivity for the TFC membranes prepared using 4 and 6 min assembly times. However, in general, in our systematic study, the ideal selectivity of the dye pairs revealed the separation potential of the membranes with sufficient accuracy. The performance of the TA-Fe^3+^ membranes for the separation of small organic molecules were compared to those from other studies in the literature, as shown in Table 2. The fabricated membranes demonstrated excellent performance considering the water permeance and selectivity in both single- and mixed-solute systems.

#### 3.2.4. Evaluation of Membrane Antifouling Performance

Membrane fouling is a key factor for the evaluation of membrane performance. The decrease in flux during process operation due to fouling results in performance decay and increases the operational cost [54]. Therefore, we further investigated the fouling property of the fabricated membranes using humic acid (HA) as a model foulant. The study was carried out periodically to assess the changes in the flux of the MPN membranes. Figure 5e displays the normalized membrane flux during different stages. The HA solution filtration was performed in a steady state. All TA-Fe^3+^ membranes showed a decline in flux during the fouling period due to the adsorption and accumulation of HA molecules on the membrane surface and pore blockage. The membrane flux recovered to a certain extent after cleaning. In the first cycle, the flux recovery ratio (FRR) was in the range of 64–78% (Figure 5f). During the second cycle, the FRR exhibited a slight decrease due to irreversible fouling on the surface of the membranes. The antifouling property of the membranes is ascribed to the hydrophilic nature of the MPN membrane surfaces. Incorporation of tannic acid in membrane surfaces or pore walls weakens the hydrophobic interaction between the membranes and foulant molecules [55]. A protective hydration layer is formed by the preferential adsorption of water molecules on the –OH-containing self-assembled film [36,56]. This inhibition of membrane fouling prevents the decline in HA solution flux. Moreover, it enhances the FRR after membrane cleaning. Surface charge also plays a key role in affecting the membrane antifouling properties. TA contains acidic catechol groups that can tune the surface property of thin films in constructing antifouling surface substrates [57]. Generally, the fabricated membranes had a negative surface potential (Figure 3a). The antifouling property is significantly enhanced by the reversible electrostatic repulsion between HA and TA.

## 4. Conclusions

In this study, we investigated the influence of the assembly time on the synthesis of MPN TFC membranes fabricated via a layer-by-layer technique from aqueous solutions of low concentrated TA and a ferric ion. By varying the assembly time between 1 and 6 min, we prepared membranes with similar surface charge densities but different porosities. This work was focused on analyzing the performance of TA-Fe^3+^ TFC membranes for the separation of neutral and anionic organic molecules, both in single-solute and mixed-solute experiments. Over the years, there has been a considerable interest in the separation of small organic molecules in the nanometer dimension due to the lack of commercially viable membranes for this purpose. To the best of our knowledge, in this work, for the first time, we have demonstrated that TFC membranes containing TA-Fe^3+^ selective layers can be used for solute–solute separation with high selectivity from aqueous solutions containing a mixture of small organic solutes. Aqueous solutions containing equimolar mixtures of the riboflavin/naphthol green B pair and the orange II/naphthol green B pair were used as feed solutions. The membranes showed a high rejection of negatively charged solutes while low rejection was observed for neutral organic solutes. The electrostatic interaction between the negatively charged membrane surfaces and the organic molecules led to the selective permeation of the neutral solute riboflavin over naphthol green B. The increase in the assembly time generally improved the selectivity of the riboflavin/naphthol green B pair while the permeance decreased owing to a loss of surface porosity. This is a general trend observed in most of the separation processes, which is often referred to as the trade-off between permeance and selectivity. The results showed that the TA-Fe^3+^ membrane fabricated at 4 min of assembly time demonstrated the best performance in terms of its high selectivity for neutral/charged dye separation and its water flux. For the orange II/naphthol green B (OR-/NGB3-) anionic dye pair, there was no trade-off between permeance and selectivity. The TFC membrane prepared using a 1 min assembly time (MPN-1) showed the highest water permeance and high selectivity for the OR-/NGB3- dye pair. The present study indicates that the self-assembled selective layers of metal–polyphenol-network-based TFC membranes obtained from a green synthesis pathway have great potential in the treatment and fractionation of a mixture of complex streams. Overall, this study proves that the assembly time is a vital tool that may be used to control the surface morphology and tune the separation performance of TFC membranes containing TA-Fe^3+^ selective layers prepared via LBL.

## Figures and Tables

**Table 1:** TA-Fe^3+^ selective layer casting parameters.

Membrane Name	Casting Solution Concentration [Weight %]		Casting Solution Concentration [mM]		Assembly Time [min]	TA Solution pH	Number of TA-Fe^3+^ Layers Deposited
	TA	FeCl_3_.6H_2_O	TA	FeCl_3_.6H_2_O			
MPN-1	0.02	0.09	0.1176	3.330	1	5.8	2
MPN-2.5	0.02	0.09	0.1176	3.330	2.5	5.8	2
MPN-4	0.02	0.09	0.1176	3.330	4	5.8	2
MPN-6	0.02	0.09	0.1176	3.330	6	5.8	2

**Table 2:** Performance comparison of the TA-Fe^3+^ membranes with other reported studies for charge- and charge/size-based separation of small organic molecules.

Membrane Type	Small Molecules	Molecular Weight (g.mol^−1^)	Molecular Charge	Selectivity Diffusion ^(a)^	Selectivity Filtration		Water Permeance (L·m^−2^·h^−1^· bar^−1^)	Reference
					Single Solutes	Mixed Solutes		
MPN-1	RB0	376.36	0	--	5.3	6.5	86.1	This work
	NGB3-	878.45	−3					
MPN-2.5	RB0	376.36	0	--	8.3	9.3	39.8	This work
	NGB3-	878.45	−3					
MPN-4	RB0	376.36	0	--	23.4	18.4	13.6	This work
	NGB3-	878.45	−3					
MPN-6	RB0	376.36	0	--	26.7	18.4	5.6	This work
	NGB3-	878.45	−3					
MPN-1	RB0	376.36	0	--	2.0	--	86.1	This work
	OR-	350.32	−1					
MPN-2.5	RB0	376.36	0	--	4.1	--	39.8	This work
	OR-	350.32	−1					
MPN-4	RB0	376.36	0	--	8.5	--	13.6	This work
	OR-	350.32	−1					
MPN-6	RB0	376.36	0		13.2	--	5.6	This work
	OR-	350.32	−1					
MPN-1	OR-	350.32	−1	--	2.9	3.6	86.1	This work
	NGB3-	878.45	−3					
MPN-2.5	OR-	350.32	−1	--	2	2.5	39.8	This work
	NGB3-	878.45	−3					
MPN-4	OR-	350.32	−1	--	2.7	1.7	13.6	This work
	NGB3-	878.45	−3					
MPN-6	OR-	350.32	−1	--	2	1.9	5.6	This work
	NGB3-	878.45	−3					
MPN 1TA-3Fe	RB0	376.36	0	--	3.2	--	62.5	[38]
	OR-	350.32	−1					
MPN 1TA-4.5Fe	RB0	376.36	0	--	8.5	--	13.6	[38]
	OR-	350.32	−1					
MPN 1TA-6Fe	RB0	376.36	0	--	20.6	--	3.8	[38]
	OR-	350.32	−1					
MPN 1TA-8Fe	RB0	376.36	0	--	3.2	--	0.9	[38]
	OR-	350.32	−1					
Amphiphilic random copolymer membrane	Riboflavin	376.36	0	263	8.4 ^(b)^	19.2 ^(b)^	4.2	[3]
	Acid blue 45	474.33	−2					
Positively charged triblock copolymer SNIPS membrane (quaternized P4VP block)	RB0	376.36	0	--	21.3	28.3	11.0	[6]
	Methylene blue	319.85	+1					
Negatively charged triblock copolymer SNIPS membrane (sulfonated)	OR-	350.32	−1	--	14.7	44.6	9.5	[6]
	NGB3-	878.45	−3					
Negatively charged triblock copolymer SNIPS membrane (sulfonated)	OR-	350.32	−1	--	64.3	-	9.5	[6]
	Reactive green 19	1418.93	−6					
Isoporous block copolymer membrane	RB0	376.36	0		35.7	39.9	3.8	[50]
	Methylene blue	319.85	+1					
Negatively charged diblock copolymer SNIPS membrane (sulfonated)	OR-	350.32	−1	--	5.2	--	74	[7]
	Reactive green 19	1418.93	−6					
NP-Den hybrid membrane	Rhodamine 6G	479.02	+1	11	--		--	[51]
	Calcein	622.53	−4					
Self-assembled polyelectrolyte deposited PCTE	Rhodamine 6G	479.02	+1	3.5	--		--	[52]
	Calcein	622.53	−4					
Cationic dendrimer deposited PCTE	Calcein	622.53	−4	10	--		--	[53]
	Rhodamine 6G	479.02	+1					

^(a)^ The selectivities were determined from a single-solute system. ^(b)^ We calculated the selectivities using the reported retention values.

## Data Availability

Data are contained within the article and Supplementary Materials.

## Supplementary Materials

The following supporting information can be downloaded at https://www.mdpi.com/article/10.3390/membranes14060133/s1, Figure S1: Sulfate ion rejection of the fabricated membranes and their respective salt solution permeance; Figure S2: Surface wettability of the pristine PAN and TA-Fe^3+^ membranes fabricated at different assembly times; Table S1: Theoretical Fe/O ratios in the three different complex states of TA-Fe^3+^ self-assembly; Table S2: Comparison of the Na_2_SO_4_ separation performance of the fabricated selective layers and the TA-M^n+^ membranes using porous supports in the literature.
