# Supplementary material for: Using the Assembly Time as a Tool to Control the Surface Morphology and Separation Performance of Membranes with a Tannic Acid–Fe3+ Selective Layer
Source: Membranes (Basel). 2024 Jun 6;14(6):133. doi: 10.3390/membranes14060133 (PMC11205845; doi:10.3390/membranes14060133)
Supplement: Supplementary file 1 [file membranes-14-00133-s001.zip › membranes-3008099-supplementary.pdf]

membranes

Supplementary information

Using the Assembly Time as a Tool to Control the Surface Morphology and Separation Performance of Membranes with a Tannic Acid–Fe^{3+} Selective Layer

Hluf Hailu Kinfu [1], Md. Mushfequr Rahman [1,*], Erik S. Schneider [1], Nicolás Cevallos-Cueva [1] and Volker Abetz [1,2]

[1] Helmholtz-Zentrum Hereon, Institute of Membrane Research, Max-Planck-Straße 1, 21502 Geesthacht, Germany; hluf.kinfu@hereon.de (H.H.K.); erik.schneider@hereon.de (E.S.S.); nicolas.cevallos-cueva@hereon.de (N.C.-C.); volker.abetz@hereon.de (V.A.)
[2] Institute of Physical Chemistry, University of Hamburg, Martin-Luther-King-Platz 6, 20146 Hamburg, Germany
* Correspondence: mushfequr.rahman@hereon.de; Tel.: +49-4152872446

Salt Rejection Performance of the TA-Fe^{3+} TFC Membranes

Evaluation of Membrane Performance: The membrane rejection towards a salt solution was evaluated with a 1 g/L Na_2SO_4 filtration experiment. We used the dead-end filtration mode of a stirred test cell from Millipore (EMD Millipore XFUF07601) at a stirring speed of 350 rpm, and a 2.13 cm^2 membrane active area was used. Membrane compaction was carried out for at least 3 h at 4 bar before rejection tests were performed at 3 bar transmembrane pressure. The concentrations of the feed, permeate and retentate samples from the rejection test of the Na_2SO_4 aqueous solution were analyzed using ion chromatography (Dionex ICS600, Thermofischer Scientific Inc., USA).

The rejection characteristics of the membranes were then evaluated as follows:

$$R\ (\%) = \left(1 - \frac{C_p}{(C_f + C_r)/2}\right) * 100 \qquad (S1)$$

where R is solute retention, and C_p, C_f and C_r are the concentrations of the permeate, concentration of the feed and concentration of retentate solutions in mg·L^{-1}, respectively. The average of the feed and retentate concentrations was taken to account for the small change in feed-side concentration in the dead-end filtration mode.

The solution permeance, P_s (L·m^{-2}·h^{-1}·bar^{-1}), was calculated using the following equation:

$$P_s = \frac{V}{A * \Delta t * \Delta P} \qquad (S2)$$

where V (L) is the volume of permeate collected, A (m^2) is the effective area of the membrane, Δt (h) is the filtration time and ΔP (bar) is the applied transmembrane pressure.

Results and Discussion

The ion rejection performance of the membranes is presented in Figure S1. Sulfate rejection by the PAN support was only 2.8 ± 1 %, which demonstrated that ions can easily permeate the porous support. The rejection of sulfate was notably higher in the self-assembled TFC membranes than the pristine support. Consistent with the large pore sizes,

the TA-Fe^{3+} membranes synthesized at 1 min (MPN-1) and 2.5 min (MPN-2.5) showed relatively low rejection of Na_2SO_4. However, when the assembly time was increased, the pore size was reduced significantly, which further contributed to the enhanced rejection of SO_4^{2-} ions. The membranes fabricated at coating times of 4 min (MPN-4) and 6 min (MPN-6) had 90.4% and 97.3% sulfate rejection, respectively. Salt rejection is mainly affected by steric hindrance, dielectric exclusion and electrostatic repulsive effects [1-3]. Sulfate is a divalent anion that can be highly rejected by negatively charged surfaces due to the Donnan exclusion effect, and strong electrostatic repulsion between the negatively charged TA-Fe^{3+} surface and SO_4^{2-} anions contributes to the overall rejection of the membranes. However, as the TFC membranes showed similar surface zeta potentials (Figure 2e in the main manuscript), the differences in rejection for the membranes fabricated at different assembly times are attributed to the change in pore size, a variation that affects both steric and dielectric exclusion. The latter stems from a decreased dielectric constant of the solvent inside small confined pores and the developed solvation energy barrier [4].

A comparison of the salt retention performance levels of the membranes with those of other tannic acid–metal ion membranes published in the literature is provided in Table S2.

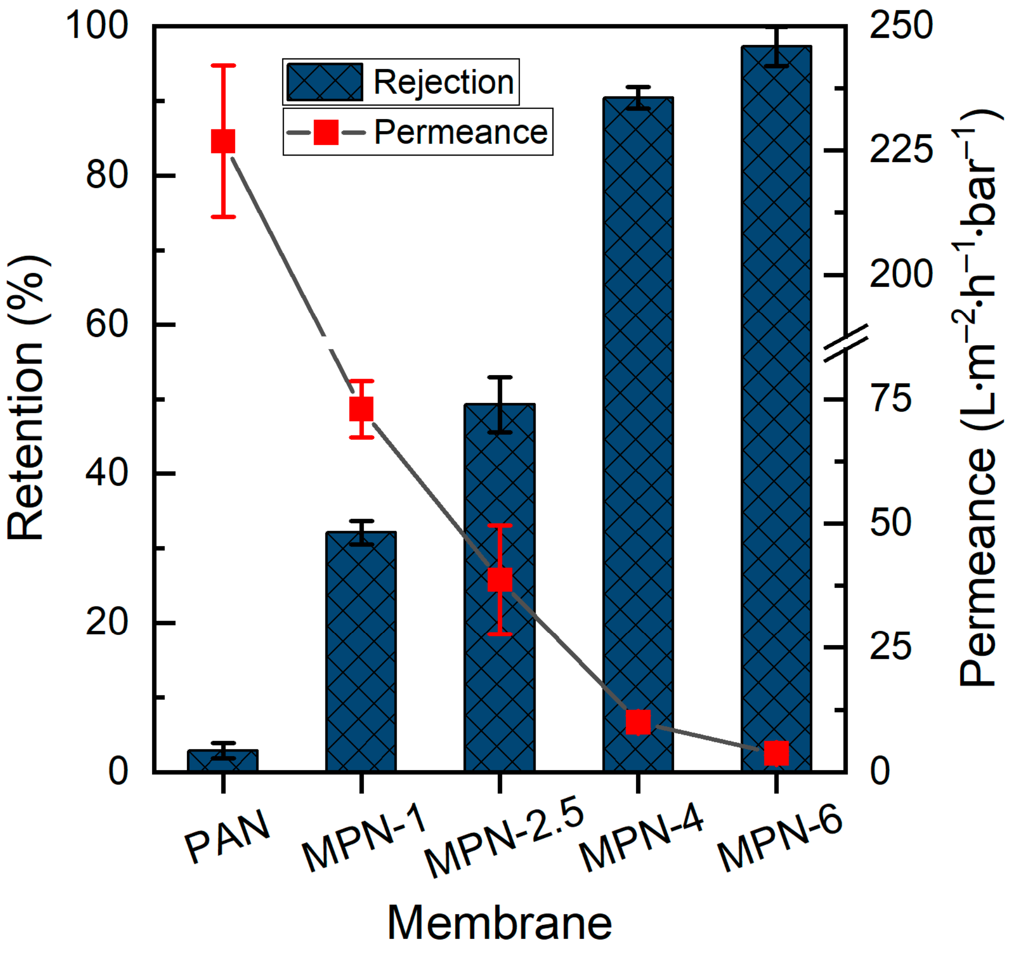

Figure S1. Sulfate ion rejection of the fabricated membranes and their respective salt solution permeance. A salt solution of 1 g/L Na_2SO_4 was used as a feed solution. The solid line is added to guide the eye.

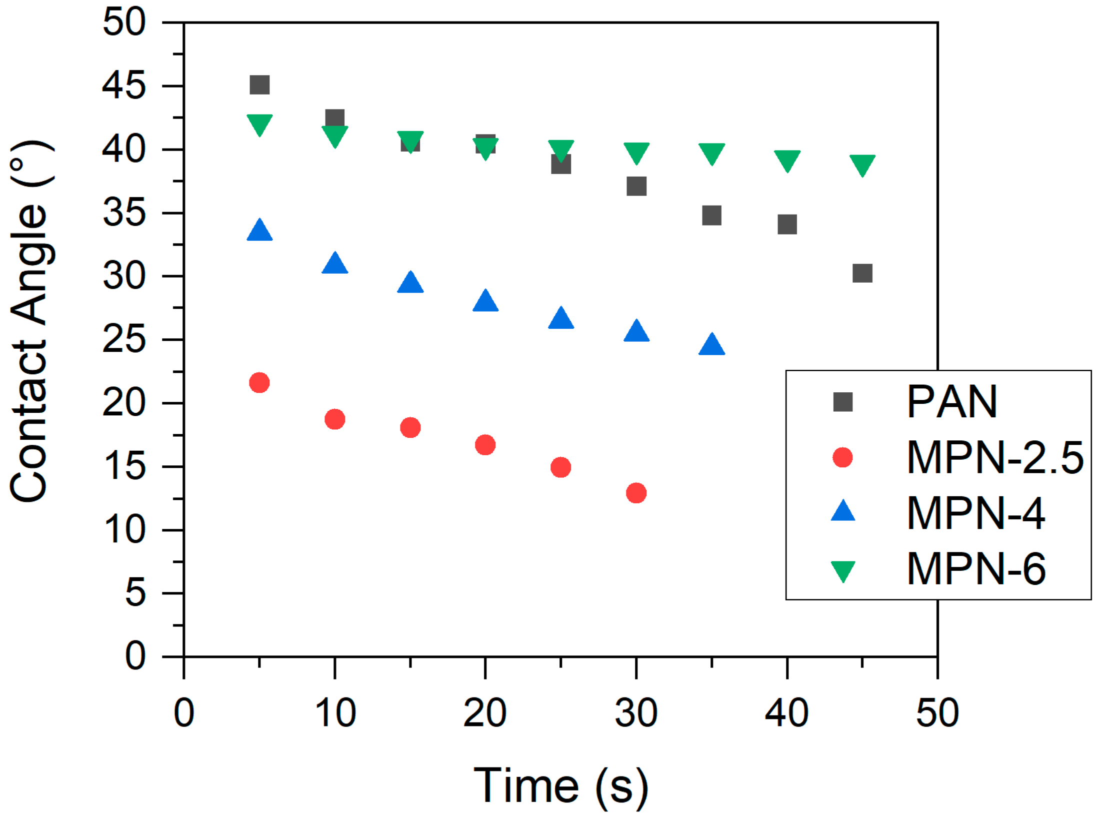

Figure S2. Surface wettability of the pristine PAN and TA-Fe^{3+} membranes fabricated at different assembly times.

Table S1. Theoretical Fe/O ratios in the three different complex states of TA-Fe^{3+} self-assembly. Although TA contains 46 oxygens in its elemental structure, only 25 from the hydroxyl groups of catechol and galloyl moieties can coordinate with metal ion centers.

Complexation state	Number of ligands and metal ions present in the complex	Total oxygen in the complex state	Number of total OH groups capable of coordination	Number of OH groups coordinating with 1 Fe center	Fe centers required for total coordination of available (capable) OH groups	Fe/O ratio in the complex state
Mono-complex	1TA-1Fe	46	25	2	12.5	0.27
Bis-complex	2TA-1Fe	92	50	4	12.5	0.14
Tris-complex	3TA-1Fe	138	75	6	12.5	0.09

Table S2. Comparison of the Na_2SO_4 separation performance of the fabricated selective layers and the TA-M^{n+} membranes using porous supports in the literature.

Membranes	Synthesis Method	Assembly time	Pure water permeance ($L \cdot m^{-2} \cdot h^{-1} \cdot bar^{-1}$)	Na_2SO_4 rejection (%)	Operating conditions	Ref.
TA−Fe^{3+}/PES	Co-deposition	1 min	5.5	89.7	3.4 mM Na_2SO_4 at 3 bar	[5]
TA−Fe^{3+}/PAN−COOH	Co-deposition	2 min	13.6	90.2	1 g/L Na_2SO_4 at 0.5 MPa	[6]
TA−Fe^{3+}/PAN	LBL	1 min	40.9	15.0	dye/ Na_2SO_4 solution at 0.2 MPa	[7]
TA−Fe^{3+}/PAN	Co-deposition	1 min	14.2	87.6	1 g/L Na_2SO_4 at 3 bar	[8]
TA−Cu^{2+}/PAN	Co-deposition	10 min	52	17	-	[9]
TA−Fe^{3+}/PES	Co-deposition	1 min	17.2	62.1	1 g/L Na_2SO_4 at 0.2 MPa	[10]
TA−Ti^{4+}/PSf	Co-deposition	9 h	9.5	70.3	5 mM Na_2SO_4 at 0.2 MPa	[11]
TA−Fe^{3+}/PAN−COOH	Contra-diffusion	7 min	7.4	95.6	1 g/L Na_2SO_4 at 25 °C and 0.6 MPa	[12]
TA−Fe^{3+}/PAN	LBL	2.5 min	39.8	49.3	1 g/L Na_2SO_4 at 3 bar	This work
		4 min	13.5	90.4	1 g/L Na_2SO_4 at 3 bar	This work
		6 min	5.6	97.3	1 g/L Na_2SO_4 at 3 bar	This work

* TA-M^{n+}, tannic acid−metal ion; PAN, polyacrylonitrile; PAN−COOH, hydrolyzed polyacrylonitrile; PSf, polysulfone; PES, polyethersulfone; LBL, layer by layer

References

1. Escoda, A.; Deon, S.; Fievet, P. Assessment of dielectric contribution in the modeling of multi-ionic transport through nanofiltration membranes. *Journal of Membrane Science* **2011**, *378*, 214-223.
2. Fievet, P. Donnan steric pore (dsp) and dielectric exclusion (de) model. In *Encyclopedia of membranes*, Drioli, E.; Giorno, L., Eds. Springer Berlin Heidelberg: Berlin, Heidelberg, 2015; pp 1-5.
3. Szymczyk, A.; Fievet, P. Investigating transport properties of nanofiltration membranes by means of a steric, electric and dielectric exclusion model. *Journal of Membrane Science* **2005**, *252*, 77-88.
4. Oatley, D.L.; Llenas, L.; Aljohani, N.H.M.; Williams, P.M.; Martinez-Llado, X.; Rovira, M.; de Pablo, J. Investigation of the dielectric properties of nanofiltration membranes. *Desalination* **2013**, *315*, 100-106.
5. Guo, H.; Peng, L.E.; Yao, Z.; Yang, Z.; Ma, X.; Tang, C.Y. Non-polyamide based nanofiltration membranes using green metal-organic coordination complexes: Implications for the removal of trace organic contaminants. *Environmental Science and Technology* **2019**, *53*, 2688-2694.
6. Liu, D.; Chen, Y.; Tran, T.T.; Zhang, G. Facile and rapid assembly of high-performance tannic acid thin-film nanofiltration membranes via fe3+ intermediated regulation and coordination. *Separation and Purification Technology* **2021**, *260*, 118228-118228.
7. Xiao, Y.; Guo, D.; Li, T.; Zhou, Q.; Shen, L.; Li, R.; Xu, Y.; Lin, H. Facile fabrication of superhydrophilic nanofiltration membranes via tannic acid and irons layer-by-layer self-assembly for dye separation. *Applied Surface Science* **2020**, *515*, 146063-146063.
8. Peng, L.E.; Yao, Z.; Chen, J.; Guo, H.; Tang, C.Y. Highly selective separation and resource recovery using forward osmosis membrane assembled by polyphenol network. *Journal of Membrane Science* **2020**, *611*, 118305-118305.
9. Chakrabarty, T.; Pérez-Manríquez, L.; Neelakanda, P.; Peinemann, K.V. Bioinspired tannic acid-copper complexes as selective coating for nanofiltration membranes. *Separation and Purification Technology* **2017**, *184*, 188-194.
10. Fan, L.; Ma, Y.; Su, Y.; Zhang, R.; Liu, Y.; Zhang, Q.; Jiang, Z. Green coating by coordination of tannic acid and iron ions for antioxidant nanofiltration membranes. *RSC Advances* **2015**, *5*, 107777-107784.
11. Wu, H.; Xie, J.; Mao, L. One-pot assembly tannic acid-titanium dual network coating for low-pressure nanofiltration membranes. *Separation and Purification Technology* **2020**, *233*, 116051-116051.
12. Wang, Y.X.; Zhu, C.Y.; Lu, F.; Yu, Z.F.; Yang, H.C.; Xue, M.; Xu, Z.K. Metal-polyphenol coordination at the aqueous contra-diffusion "interface": A green way to high-performance iron(iii)/tannic acid thin-film-composite nanofiltration membranes. *Langmuir* **2022**.

Disclaimer/Publisher's Note: The statements, opinions and data contained in all publications are solely those of the individual author(s) and contributor(s) and not of MDPI and/or the editor(s). MDPI and/or the editor(s) disclaim responsibility for any injury to people or property resulting from any ideas, methods, instructions or products referred to in the content.
